# Frontotemporal dementia: An updated overview

**Published:** 2009-01

**Authors:** E. Mohandas, V. Rajmohan

**Affiliations:** Department of Psychiatry, Elite Mission Hospital, Koorkkencherry, Thrissur, Kerala, India

**Keywords:** Frontotemporal dementia, frontotemporal lobar degeneration, progressive nonfluent aphasia, neurodegenerative disorders

## Abstract

Frontotemporal dementia (FTD) is a progressive neurodegenerative syndrome occurring between 45 and 65 years. The syndrome is also called frontotemporal lobar degeneration (FTLD). However, FTLD refers to a larger group of disorders FTD being one of its subgroups. The other subgroups of FTLD are progressive nonfluent aphasia (PFNA), and semantic dementia (SD). FTLD is characterized by atrophy of prefrontal and anterior temporal cortices. FTD occurs in 5-15% of patients with dementia and it is the third most common degenerative dementia. FTD occurs with equal frequency in both sexes. The age of onset is usually between 45 and 65 years though it may range anywhere from 21 to 81 years. The usual course is one of progressive clinicopathological deterioration with mortality within 6-8 years. Unlike Alzheimer’s disease (AD), this condition has a strong genetic basis and family history of FTD is seen in 40-50% of cases. FTD is a genetically complex disorder inherited as an autosomal dominant trait with high penetrance in majority of cases. Genetic linkage studies have revealed FTLD loci on chromosome 3p, 9, 9p, and 17q. The most prevalent genes are PGRN (progranulin) and MAPT (microtubule-associated protein tau), both located on chromosome 17q21. More than 15 different pathologies can underlie FTD and related disorders and it has four major types of pathological features: (1) microvacuolation without neuronal inclusions, (2) microvacuolation with ubiquitinated rounded intraneuronal inclusions and dystrophic neurites FTLD-ubiquitinated (FTLD-U), (3) transcortical gliosis with tau-reactive rounded intraneuronal inclusions, (4) microvacuolation and taupositive neurofibrillary tangles. Behavior changes are the most common initial symptom of FTD (62%), whereas speech and language problems are most common in NFPA (100%) and SD (58%). There are no approved drugs for the management of FTD and trials are needed to find effective agents. Non-pharmacological treatment and caregiver training are important in the management of FTD.

## INTRODUCTION

Frontotemporal dementia (FTD), with its initial description as Pick’s disease (1892) encompasses a group of progressive neurodegenerative syndromes. Three common syndromes of FTD include frontal variant of FTD (fvFTD) or behavioral variant of FTD (bvFTD), progressive nonfluent aphasia (PFNA), and semantic dementia (SD). FTD spectrum may also include corticobasal degeneration (CBD), progressive supranuclear palsy (PSP), apraxia of speech (AOS, lumped under PFNA by some researchers), and motor neuron disease (MND) [Table 1].[1] Histopathological and genetic classification of FTD suggest the complexity of this neurodegenerative disease.[2–4]

FTD occurs in 5-15% of patients with dementia and is the third most common degenerative dementia, following only Alzheimer’s disease (AD) and dementia with Lewy bodies. FTD occurs with equal frequency in both sexes (some studies show a male preponderance). The age of onset is usually between 45 and 65 years though it may range from 21 to 81 years. The course is progressive with mortality within 6-8 years. Unlike AD, the genetic loading is more (40-50%). The early clinical picture in most cases is dominated by behavioral symptoms with cognitive symptoms appearing much later. However, the presentation may vary in PFNA and SD.[5]

**Table 1:** Classification of FTD

Frontotemporal dementia (FTD)
Subgroups
Frontal/behavioral variant of frontotemporal dementia (fvFTD/ bvFTD)
Progressive nonfluent aphasia (PFNA)
Semantic dementia (SD)
Spectrum disorders
Corticobasal degeneration (CBD)
Progressive supranuclear palsy (PSP)
Apraxia of speech (AOS)
Motor neuron disease (MND)

## GENETICS OF FTD

FTD is a genetically complex disorder inherited as an autosomal dominant trait with high penetrance in majority of cases. Genetic linkage studies have revealed FTLD loci on chromosome 3p, 9, 9p, and 17q. The most prevalent genes are PGRN (progranulin) and MAPT (microtubule-associated protein tau), both located on chromosome 17q21. The autosomal dominant form of FTLD linked to chromosome 17q21 is termed FTDP-17. The mutations in the MAPT and PGRN gene among these families are designated FTDP-17 (MAPT) and FTDP-17 (PRGN), respectively. FTD with MAPT mutations is tau-positive whereas the more common FTDP-17 (PGRN) is tau-negative.[6]

The PGRN gene encoding the PGRN protein has more than 30 mutations. PGRN, expressed in neurons and activated microglia, is involved in tissue remodeling by activating signaling cascades that control cell cycle progression and cell motility. PGRN mutations occur in 26% of familial FTD cases. PGRN mutation is associated with the expression of truncated and hyperphosphorylated isoforms of TDP-43 (TAR DNA binding protein 43). Under pathologic conditions, TDP-43 relocates from the neuronal nucleus to the cytoplasm resulting in loss of TDP-43 nuclear functions. However, the exact role and the mechanism underlying the formation of TDP-43 are still under scrutiny.[7]

MAPT having 37 mutations within the microtubule-binding region or exon 10 produce tau isoforms with either three microtubule-binding repeats (3R-tau) or four repeats (4R-tau). In Pick’s disease 3R-tau accumulates. The common tau substitutions associated with FTDP-17, P301L, N279K, and a splice site mutation (exon10 +16), account for around 60% of cases.[8] Missense and deletion mutations disrupts the binding of tau to microtubules resulting in accumulation of unbound tau. Alternative splicing of exon 10 leads to disruption of 3R-tau: 4R-tau ratio.[7]

MAPT mutation carrier phenotypes demonstrate behavioral changes, dementia, and parkinsonism, with an average disease duration of seven years. MAPT mutations on exons 1, 9, and 11 to 13 account for the dementia-dominant phenotype. The parkinsonism-plus-predominant phenotype is associated with mutations within intron and exon 10, leading to the overproduction of 4R-tau isoforms.[8] FTD linked to chromosome 3 (FTD3) is associated with aberrant splicing of CHMP2B (chromatin-modifying protein 2B) gene. This uncommon type of FTD is characterized by global cortical atrophy with no distinctive histopathologic features.

Six missense mutations in VCP (Valosin-containing protein) gene is identified in IBMPDF (inclusion body myopathy associated with Paget disease of bone and FTD). VCP gene codes for the protein VCP, which is a member of the AAA-ATPase (ATPases associated with diverse cellular activities) superfamily, and is involved in cell cycle control, membrane fusion, and the ubiquitin-proteasome degradation pathway.

Mutations in the presenilin-1 gene (PSEN1) and Lrrk2 (leucine-rich repeat kinase) G2019S substitution are other candidate genes which are being investigated in FTD. FTD-MND is linked to two separate loci, both on chromosome 9, located on the long (9q21-22) and short arms (9p13.2-21.3). The responsible genes remain to be identified but nonsense mutations in the intraflagellar transport 74 (IFT74) gene are described in one FTD-ALS family.[9]

## PATHOLOGICAL FEATURES

More than 15 different pathologies may underlie FTD and related disorders. Immunohistochemical analysis defines four major types of pathological features:

- Microvacuolation without neuronal inclusions, that is, dementia lacking distinctive histological (DLDH) features.
- Microvacuolation with ubiquitinated rounded intraneuronal inclusions and dystrophic neurites within layer 2 of frontal and temporal neocortex and hippocampal dentate gyrus cells designated FTLD-ubiquitinated (FTLD-U) type.
- Transcortical gliosis with tau-reactive rounded intraneuronal inclusions (Pick’s bodies) and swollen achromatic neurons (Pick’s cells).
- Microvacuolation and taupositive neurofibrillary tangles or Pick-like bodies in neurons, and sometimes tangles in glial cells of the cerebral cortical white matter. This is associated with familial FTD because of mutations in the tau gene. Types c and d are referred to as tauopathies.[10]

The most common pathology associated with FTD was thought to be DLDH characterized by neuronal loss, and gliosis affecting superficial cortical lamina and absence of the typical pathological findings of AD. Many DLDH now are found to be FTLD-U. Some DLDH have evidence of motor neuron degeneration and are separated from FTLD-U and called FTLD-MND. Hippocampal sclerosis is common in FTLD-U but uncommon in FTLD-MND. This subgrouping of DLDH makes the diagnosis of true DLDH extremely rare. One of the major ubiquitinated proteins in FTLD-U, FTLD-MND is TDP-43. These disorders are therefore called TDP-43 proteinopathies and majority of FTLD cases are TDP-43 proteinopathies. The other significant groups of FTLD are characterized by the presence of MAPT and are called tauopathies. More than 90% of cases of FTD and related disorders can be classified as TDP-43 proteinopathy or tauopathy [Table 2]. A small proportion is not characterized by either abnormal TDP-43 or tau. The pathology in them include neurofilament inclusion body disease (characterized by intraneuronal inclusions that are immunoreactive to neurofilamentand α-internexin) and basophilic inclusion body disease.[11]

**Table 2:** Pathologic classification of FTD and related disorders

Tauopathies	TDP 43 Proteinopathies O	Others
Pick’s disease	FTLD-U Type 1	DLDH
Progressive supranuclear palsy	FTLD-U Type 2	Neurofilament inclusion body disease
Corticobasal degeneration	FTLD-U Type 3 (PGRN mutation)	Basophilic inclusion body disease
ALS-Parkinsonism complex of Guam	FTLD-U Type 4 (VCP mutation)	FTLD+ CHMP 28
Sporadic multisystem tauopathy	FTLD- primary lateral sclerosis	FTLD-U non-TDP 43
Argyrophilic grain disease		
Tangle dominated dementia		
Diffuse neurofibrillary tangle dementia with calcifications		

## NEUROIMAGING IN FTD

FTD generally exhibits atrophy in the frontal and temporal lobes. The ventricles may be enlarged and the head of the caudate may be atrophic. Depending on the stage, the atrophy may be subtle to severe in affected regions and in the later stages may produce the so-called ’’knife blade’’ appearance of the affected gyri.[12] The atrophy may be asymmetric, a feature characteristic of primary progressive aphasia and semantic dementia. Volumetric analysis of different regions may be useful in discriminating FTD from AD. The three main FTD syndromes, bvFTD, SD and PNFA, have somewhat distinct patterns of atrophy. The bvFTD is associated with atrophy affecting bilateral frontal lobes, particularly the medial frontal lobes, and anterior temporal lobes, whereas SD is associated with bilateral, although usually asymmetric, middle, inferior, and medial anterior temporal lobe atrophy. PNFA shows left perisylvian atrophy.[13]

Functional imaging studies such as single-photon computed tomography (SPECT) and positron emission tomography (PET) especially recent studies using SPECT or FDG-PET in bvFTD highlight in particular the involvement of the medial prefrontal cortex, and to a variable degree the posterior orbitofrontal/subcallosal cortex, dorsolateral prefrontal regions and insula. In addition, some patients may also show changes in basal ganglia and thalamus. Studies examining regional metabolic changes and behavioral scores, typically focusing on apathy and disinhibition found that damage to the orbitofrontalcortex is associated with both clinical features.[14]

Imaging in FTD related conditions show distinctive findings. Posterior frontal and superior parietal atrophy is associated with CBS, whereas those who have PSP show areas of subcortical atrophy affecting the superior cerebellar peduncle and midbrain. The anterior-posterior diameter of the midbrain is reduced, giving the so-called ’’face of Mickey mouse’’ appearance. Patients who have AOS show atrophy of the supplemental motor area and superior posterior frontal lobe.[14]

## CLINICAL FEATURES

Behavior changes are the most common initial symptom of FTD (62%), whereas speech and language problems are most common in NFPA (100%) and SD (58%). About 10% of patients with FTD present with memory problems as a first symptom. Common behavioral symptoms in FTD include apathy (32%) and disinhibition (16%). Most patients with SD begin with language (left-sided cases) or emotional (right-sided cases) changes. The lack of insight seen in FTD, and sometimes in SD, leads patients to ignore or deny their deficits often delaying diagnosis [Table 3]. Insight is usually preserved in NFPA.[15]

**Table 3:** Differentiating among FTLDs

	FTD	SD	PFNA
Sex	Male=female (M>F)	Male>female	Female>male
Age of onset	Mid 50s	Late 50s	Early 60s
Genetics	Strongly familial	Rarely familial	Intermediate
MND	Common	Unusual	Unusual
Behavior	Personality changes, apathy, disinhibition, loss of insight	Similar to FTD in right sided cases; FTD-type behaviors emerge after a few years in left-sided cases	Tends to remain normal; depression is common
Neurology	Look for ALS, Parkinsonian features Setshifting, inhibition; good drawing, poor generation naming	Look for ALS, often normal Poor naming, verbal memory; good working memory	Overlap with PSP and CBDNonfluent, verbal apraxia; good comprehension
Neuropsychology	Setshifting, inhibition; good drawing, poor generation naming	Poor naming, verbal memory; good working memory	Nonfluent, verbal apraxia; good comprehension
Neuroimaging	B/ (R > L) frontal–ventral–insular and cingulated atrophy/ hypometabolism (L>R) anterior temporal, amygdala and insular atrophy/hypometabolism	Bilateral	Bilateral (left > right) fronto-insular atrophy/ hypometabolism
Neuropathology	Most common: FTD ubiquitin inclusions; less common: no inclusions or tau inclusions	Most common pathology is FTD-ubiquitin inclusions	Most common pathology is FTD-tau; often overlaps with CBD or PSP

Differentiating among FTLDs (adapted from Wang P-N and Miller BL, 2007)

Clinical and pathological diagnostic criteria for FTD were initially developed by the Lund and Manchester groups. Later McKhann and colleagues proposed simpler guidelines that would facilitate easy recognition of the various FTD syndromes. The six proposed criteria are: (1) early and progressive change in personality or language; (2) impairment in social or occupational functioning; (3) a gradual and progressive course; (4) exclusion of other causes; (5) presence of deficits in the absence of delirium; and (6) exclusion of psychiatric causes such as depression. Neary criteria recognized three clinical syndromes and used the term frontotemporal lobar degeneration (FTLD) to incorporate them. The core features of FTD as defined by the Neary criteria are early decline in social and personal conduct, emotional blunting, and loss of insight [Table 4]. The clinical picture of early behavioral manifestations followed by cognitive symptoms in later stage, the gross and histopathologic features distinguishes FTD from AD[2] [Table 5].

**Table 4:** Consensus criteria for FTD

**Core diagnostic features** A. Insidious onset and gradual progression B. Early decline in social interpersonal conduct C. Early impairment in regulation of personal conduct D. Early emotional blunting E. Early loss of insight **Supportive diagnostic features** **A. Behavioral disorder** 1. Decline in personal hygiene and grooming 2. Mental rigidity and inflexibility 3. Distractibility and impersistence 4. Hyperorality and dietary changes 5. Perseverative and stereotyped behavior 6. Utilization behavior **B. Speech and language** 1. Altered speech output a. Aspontaneity and economy of speech b. Pressure of speech 2. Stereotypy of speech 3. Echolalia 4. Perseveration 5. Mutism **C. Physical signs** 1. Primitive reflexes 2. Incontinence 3. Akinesia, rigidity, and tremor 4. Low and labile blood pressure **D. Investigations** 1. Neuropsychology: impairment on frontal lobe tests without severe amnesia, aphasia, or perceptuospatial disorder 2. Electroencephalography: normal on conventional EEG despite clinically evident dementia 3. Brain imaging (structural and/or functional): predominant frontal and/or anterior temporal abnormality

Consensus criteria for FTD (based on Neary, 1998)[5]

**Table 5:** Differentiating AD and FTD

AD	FTD
Clinical features	Clinical features
Early changes	Early changes
Amnesia	Behavioral changes
Visuospatial disturbance	Stereotypy of speech
Acalculia	Kluver-Bucy (B/L Temporal)
Anomia	Language functions (SD)
Late changes	Late changes
Personality change	Amnesia
Kluver-Bucy	Visuospatial disturbance
Decreased auditory comprehension	Acalculia
Gross pathology	Gross pathology
Posterior hemispheric atrophy	Anterior hemispheric atrophy
Neurotransmitter involvement	Neurotransmitter involvement
Predominantly cholinergic/glutamatergic system	Predominantly serotonergic/ and probably dopaminergic

## MANAGEMENT

There are no specific pharmacological therapies approved for use in any of the FTD syndromes. Pharmacological interventions for the behavioral disturbances seen in FTD have included antipsychotics, antidepressants, anticonvulsants, and dopamine agonists. Behavioral impairments, such as depression, irritability, and apathy, with relative preservation of memory are compatible with serotonergic dysfunction. The majority of studies have shown deficiencies in the serotonergic system in FTD. This serotonergic deficit appears to be more postsynaptic than presynaptic. There is decreased 5-HT2A receptor density in the orbitofrontal, frontal medial, and cingulate cortices of patients. They also have decreased 5-HT1A and 5-HT2A receptors.[15] However, early trials with selective serotonin reuptake inhibitors have been equivocal or have shown moderate benefit. Recent studies of serotonin-specific reuptake inhibitors (SSRIs) and the antidepressant trazodone have shown promising results for management of behavioral disturbance in FTD. Rivastigmine has been reported to be more efficacious than galantamine and donepezil in open label data and small randomized trials.[16]

The food and drug administration (FDA) has put a black box warning label on the use of atypical antipsychotics in the treatment of elderly patients with dementia because of controversial data suggesting an increased risk of mortality.[16]

Nonpharmacological therapeutic approaches are important in the management of patients with FTD. Safety is a paramount issue, as poor judgment is often exhibited early in the course of FTD and removal of disabling firearms or dangerous power tools is appropriate. Driving safety becomes an issue as the disease progresses. Patients with poor judgment should not drive but some patients with primary progressive aphasia can drive safely. Patients who continue to drive should have periodic driving evaluations.[16]

Management of FTD concentrates mainly on the construction of a support network through social, psychiatric, and voluntary services and ultimately residential care, to relieve the immense burden on families. Education of patients and family members about the progressive nature of these conditions is important.[5] Caregiver burden increases with the progression of the disease, programs that provide respite such as daycare centers or companion care may allow the patient to remain in their home setting while allowing for the needs of the caregiver. Services are often best provided by psychiatry services for elderly people, although the existence of such services and access to them may be limited for people in dire need.[17]

## CONCLUSION

Frontotemporal dementia (FTD) still remains a poorly understood disorder. Data from genetic, histopathologic, and newer imaging techniques reveal the complex nature of this disorder. No specific pharmacological option exists although serotonin-specific reuptake inhibitors may provide modest benefit. Further research may unravel new options in the management of FTD.
